# Food allergy and anaphylaxis – 2043. Poor dilutional linearity of food allergen specific IgE measurement by immunocap in samples with high total IgE

**DOI:** 10.1186/1939-4551-6-S1-P128

**Published:** 2013-04-23

**Authors:** Maaz Mohiuddin, Michael O'Sullivan, Patricia Merkel, Cynthia Ridge, Donald Leung, Vijaya Nagabhushanam

**Affiliations:** 1 Department of Pediatrics Division of Allergy Immunology, National Jewish Health, Denver, CO, USA; 2 Department of Immunology, Pathwest, Perth, Australia; 3 Advanced Diagnostics Laboratory, National Jewish Health, Denver, CO, USA; 4 Department of Medicine Division of Pathology, National Jewish Health, Denver, CO, USA

## Background

In order to minimize the effect of non-specific IgE binding when checking for specific IgE (sIgE) to foods, it is recommended that automatic dilutions should be performed on samples with total IgE (tIgE) >20,000 kU/L to avoid false positives. We sought to determine the linearity of specific IgE measurement in serially-diluted samples with total IgE >1000 kU/L.

## Methods

31 deidentified subjects with tIgE 1000-5000 kU/L (Group A) and 10 subjects with tIgE >5000 kU/L (Group B) had sIgE measurement for egg, milk, wheat, peanut, and soybean by ImmunoCap from undiluted and 4 serial two-fold dilutions using manufacturer supplied diluent. Myeloma IgE was prepared at an initial dilution of 1:28 and then 7 serial two-fold dilutions were performed. tIgE percentage recovery was calculated by multiplying measured tIgE by the dilution factor for that sample and dividing this result by the measured tIgE in neat serum. The same calculation was applied to sIgE.

## Results

sIgE was detectable in varying concentrations for all foods in the myeloma sample and remained detectable across a range of dilutions. For Group A, mean tIgE recovery from diluted samples ranged from 120% (1:2 dilution) to 131% (1:16 dilution). Egg sIgE was the only analyte for which mean recovery was <100%. Mean percentage recovery was increased significantly for peanut, soy, and wheat (one sample t-test, p<0.05). The effect of serial dilutions on sIgE recovery for Group B was more variable.

## Conclusions

Our results raise potential issues with sIgE measurement in the setting of high tIgE as sIgE was detected in all myeloma samples which should have no allergen-specific reactivity. In Group A, there is a trend for over-recovery with serial dilutions for all allergens except egg. The variability of percentage recovery between different allergens and also between different subjects for the same allergen strongly suggests both sample and assay-specific factors are contributing to these findings. It is therefore important for laboratories and clinicians be aware of the potential limitations of sIgE measurements in patients with tIgE >1000 kU/L. Laboratories should evaluate the linearity of their own assay before routinely measuring and reporting sIgE results from diluted samples.

